# Effect of postoperative analgesia technique on the prognosis of gastric cancer: a retrospective analysis

**DOI:** 10.18632/oncotarget.21979

**Published:** 2017-10-20

**Authors:** Seokyung Shin, Hyoung-Il Kim, Na Young Kim, Ki-Young Lee, Dong Wook Kim, Young Chul Yoo

**Affiliations:** ^1^ Department of Anesthesiology and Pain Medicine, Severance Hospital, Anesthesia and Pain Research Institute, Yonsei University College of Medicine, Seodaemun-gu, Seoul 03722, Korea; ^2^ Department of Surgery, Yonsei University College of Medicine, Seodaemun-gu, Seoul 03722, Korea; ^3^ Department of Policy Research Affairs, National Health Insurance Service Ilsan Hospital, Ilsan-donggu, Goyang-si, Gyeonggi-do 10444, Korea

**Keywords:** analgesia, epidural, analgesia, patient controlled, gastric cancer

## Abstract

**Background:**

Whether regional analgesia techniques have favorable impact on prognosis after cancer surgery is unclear, and existing reports show controversial results. The aim of the present study was to evaluate and compare recurrence and mortality between patients that received either intravenous (IV) or epidural patient controlled analgesia (PCA) for pain control after curative surgery for gastric cancer.

**Materials and methods:**

Medical records of patients that underwent curative gastrectomy for gastric cancer between November 2005 and December 2010 were reviewed. Identified patients were categorized according to the use of IV or epidural PCA for postoperative analgesia. Demographic and perioperative variables including type of PCA were analyzed by univariate and multiple regression analysis to investigate any association with recurrence and mortality after surgery. Propensity score matching was done to adjust for selection bias.

**Results:**

Of the 3,799 patients included in this analysis, 374 and 3, 425 patients received IV and epidural PCAs, respectively. No difference in recurrence (HR, 1.092; 95% CI 0.859 to 1.388; P = 0.471) or mortality (HR, 0.695; 95% CI 0.429 to 1.125; P = 0.138) was identified between the use of IV and epidural PCA. Propensity score matching also showed no difference in recurrence (HR, 1.098; 95% CI 0.756 to 1.596; P = 0.623) or mortality (HR, 0.855; 95% CI 0.391 to 1.869; P = 0.695) between the two groups.

**Conclusions:**

Postoperative use of epidural analgesia was not found to be associated with reduced recurrence or mortality after curative surgery in gastric cancer patients. This finding needs to be confirmed with prospective studies in the future.

## INTRODUCTION

The notion that perioperative regional anesthesia and analgesia may improve cancer prognosis first emerged roughly a decade ago, [1–3] and was met by genuine enthusiasm of the anesthesia society. However, a decade later, we are still at a shortage of meaningful evidence to either support or refute this hypothesis. The difficulty of performing a large scale randomized controlled trial (RCT) with a relatively long follow-up period has led us to first look at the available data, and we have been presented with a number of retrospective [1, 2, 4–15] and very few prospective [6, 16] studies done on the effect of regional anesthesia and/or analgesia on the prognosis of different types of cancer.

Among several regional techniques, epidural analgesia is the most commonly employed method for operations performed in the abdominopelvic region. While there is relative abundance of retrospective and observational studies done on the effect of epidural analgesia in prostate [1, 5, 6, 8, 17–20] and colorectal cancer, [12, 13, 21–24] there are only a few that investigate gastric cancer, [4, 14, 15, 25] despite it being the fifth most common cancer worldwide. Moreover, not only do the existing studies report inconsistent results, most are based on the data of an insufficient number of a rather heterogeneous group of patients. The primary goal of this retrospective study was to compare the effect of postoperative epidural patient-controlled analgesia (PCA) vs. intravenous (IV) PCA, on recurrence and mortality in patients that received curative surgery with homogeneous surgical treatment at a high-volume center.

## RESULTS

### Study population, demographic data and perioperative characteristics

**Figure 1:**
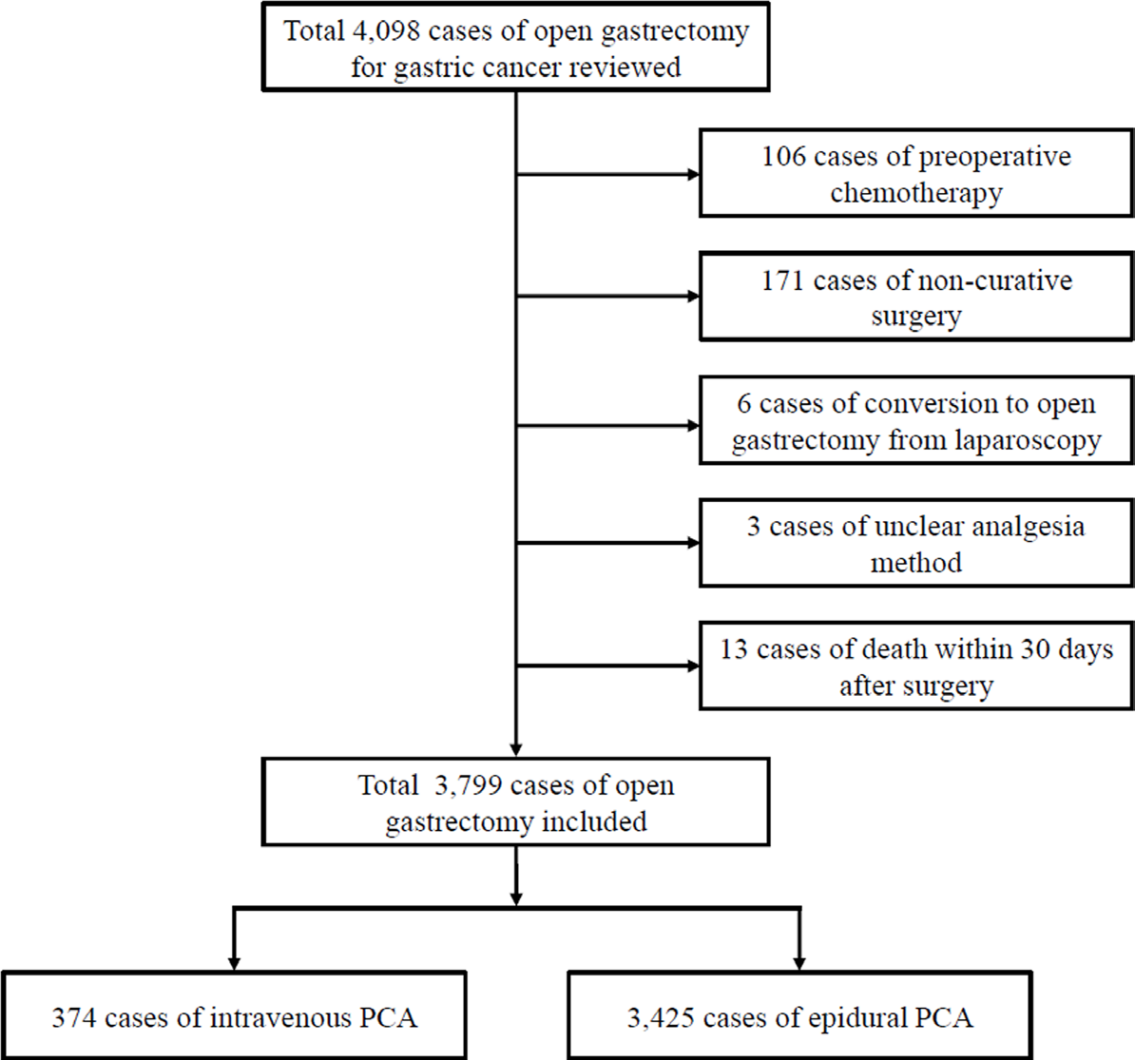
Flowchart of patient sample selection

Figure 1 shows the flowchart of patient sample selection. The data of 4, 098 patients were reviewed for analysis. Among these patients, 106 cases that received preoperative chemotherapy and 171 cases that underwent non-curative surgery were excluded. Additionally, 6 cases of laparoscopic gastrectomy that were converted to laparotomy, 3 cases of unclear analgesia method, and 13 cases of patient death within 30 days after surgery were excluded from analysis.

The remaining 3, 799 cases with 374 in the IV PCA group and 3, 425 in Epidural PCA group were compared and analyzed. The opioid used in both type of PCAs was fentanyl, and all epidural PCAs contained ropivacaine. Ropivacaine was infused at a concentration of 0.15% and mixed with fentanyl at a dose of between 2–4 μg/mL at basal rates of 4–5 mL/hr, bolus doses of 1–2 mL, and lock-out times of 15 minutes. All patients were anesthetized with balanced anesthesia which consisted of the use of a volatile anesthetic and remifentanil infusion at rates between 0.1–0.3 mcg/kg/min. Demographic characteristics of the patients are listed in Table 1. When comparing the demographic characteristics between the two groups, there was no difference in sex, height, weight, presence of hypertension, pulmonary, and liver diseases. However, there was a significant difference in age, and the presence of DM, cardiac, renal and neurologic diseases between the two groups. Greater cancer recurrence was seen with higher cancer stage (*P* = 0.012), but no difference was observed in tumor histology or resection method between the two groups.

**Table 1:** Demographic and perioperative characteristics

	IV PCA (*n =* 374)	Epidural PCA (*n =* 3,425)	*P*-value
Age (years)	59.6 ± 11.6	57.5 ± 11.7	0.001
Male sex, n	242 (64.7)	2,280 (66.6)	0.489
Height (cm)	163.4 ± 8.3	164.0 ± 8.6	0.187
Weight (kg)	62.8 ± 11.0	62.6 ± 10.0	0.678
Comorbidities			
HTN	108 (28.9)	916 (26.7)	0.390
DM	64 (17.1)	398 (11.6)	0.003
Pulmonary disease^*^	8 (2.1)	58 (1.7)	0.529
Cardiac disease^†^	35 (9.4)	102 (3.0)	< 0.001
Renal disease^‡^	16 (4.3)	23 (0.7)	< 0.001
Liver disease§	20 (5.3)	141 (4.1)	0.278
Neurologic disease^ǁ‖^	21 (5.6)	47 (1.4)	< 0.001
Cancer stage			0.012
I	175 (46.8)	1,867 (54.5)	
II	83 (22.2)	605 (17.7)	
III	116 (31.0)	953 (27.8)	
Tumor histology			0.052
AWD	38 (10.2)	416 (12.1)	
AMD	114 (30.5)	988 (28.8)	
APD	132 (35.3)	1126 (32.9)	
Mucinous	11 (2.9)	87 (2.5)	
SRC	61 (16.3)	716 (20.9)	
Other	18 (4.8)	92 (2.7)	
Lymphovascular invasion	130 (34.8)	1216 (35.5)	0.820
Resection method			0.241
Subtotal	265 (70.9)	2,527 (73.8)	
Total	109 (29.1)	898 (26.2)	
Anesthetic			
Desflurane	67 (17.9)	492 (14.4)	0.077
Enflurane	0 (0)	70 (2.0)	0.002
Isoflurane	87 (23.3)	1,032 (30.1)	0.006
Sevoflurane	220 (58.8)	1,825 (53.3)	0.043
N2O	14 (3.7)	439 (12.8)	< 0.001
Propofol 1%	295 (78.9)	2,608 (76.1)	0.249
Thiopental sodium	76 (20.3)	763 (22.3)	0.431
Other perioperative drugs			
Aspirin	2 (0.5)	7 (0.2)	0.220
NSAIDs	225 (60.2)	1229 (35.9)	< 0.001
Ephedrine	99 (26.5)	1,337 (39.0)	< 0.001
Phenylephrine	33 (8.8)	167 (4.9)	0.002
Norepinephrine	4 (1.1)	25 (0.7)	0.523
Esmolol	27 (7.2)	139 (4.1)	0.007
Labetalol	21 (5.6)	55 (1.6)	< 0.001
Nicardipine	25 (6.7)	77 (2.2)	< 0.001
Intraoperative fluid			
Colloid	62 (16.6)	561 (16.4)	0.941
Packed RBC	8 (2.1)	65 (1.9)	0.692
Recurrence	81 (21.7)	585 (17.1)	0.031
Mortality	90 (24.1)	641 (18.7)	0.015

Values are mean ± SD or *n* (%) of patients, HTN = hypertension, DM = diabetes mellitus AWD = adenocarcinoma, well differentiated, AMD = adenocarcinoma, moderately differentiated, APD = adenocarcinoma, poorly differentiated, SRC = signet ring cell. Pulmonary disease^*^ = History of pulmonary diseases, Cardiac disease^†^ = History of cardiac diseases other than hypertension, Renal disease^‡^ = History of renal diseases, Liver disease^ǁǁ^ = History of liver diseases, Neurologic disease^ǁ‖^ = History of neurologic diseases including cerebrovascular accidents.

Enflurane, isoflurane and N_2_O were anesthetics that were more often used in the Epidural PCA group, while sevoflurane was more commonly used in the IV PCA group. Among other perioperative characteristics, a significantly greater proportion of patients of the IV PCA group were found to receive NSAIDs, phenylephrine, esmolol, labetalol and nicardipine than the Epidural PCA group, while ephedrine was more commonly used in the Epidural PCA group. There was no difference in intraoperative administration of colloids or packed RBC. Patients were followed up after surgery once every 3 months during the first year, every 6 months for the following 2 years, and every year thereafter for the duration of the scheduled follow-up period. Cancer recurrence occurred in 81 (21.7%) patients of the IV PCA group and 585 (17.1%) patients of the Epidural PCA group, and the mean time to recurrence was 564.34 ± 404.0 and 545.2 ± 452.7 days in the IV PCA and Epidural PCA groups, respectively, with no significant difference (*P* = 0.717). Mortality occurred in 90 (24.1%) and 641 (18.7%) patients of the IV PCA and Epidural PCA groups, respectively. The mean follow-up time in all of the patients was 53.3 ± 21.7 months.

### Association between analgesia method and cancer recurrence and mortality after surgery

**Figure 2:**
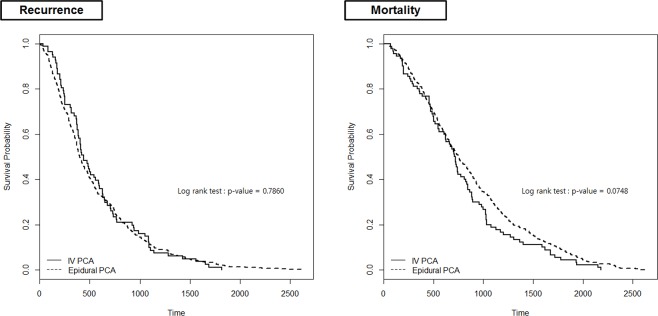
Kaplan–Meier curves for 5-year recurrence and mortality

Kaplan–Meier curves for recurrence and mortality of patients with IV PCA vs. epidural PCA are shown in Figure 2.

Neither IV nor epidural PCA was found to be associated with cancer recurrence after surgery (HR, 1.092; 95% CI, 0.859 to 1.388; *P* = 0.471) (Table 2). Factors that were related to lower recurrence were younger age and lower cancer stage. Total gastrectomy was found to be associated with greater risk of recurrence compared to subtotal gastrectomy (HR, 1.333; 95% CI, 1.132 to 1.569; *P* = 0.001). None of the anesthetics or other perioperative drugs were found to be associated with cancer recurrence. Type of postoperative PCA was also not found to be associated with mortality after open gastrectomy (*P* = 0.138) (Table 3). Other variables associated with greater mortality included older age, higher cancer stage, total gastrectomy and the administration of intraoperative phenylephrine, norepinephrine and labetalol.

**Table 2:** Univariate and multiple regression analysis of variables associated with recurrence after open gastrectomy for gastric cancer

	Univariate Analysis		Multiple Analysis	
	Hazard Ratio (95% CI)	*P*-value	Hazard Ratio (95% CI)	*P*-value
PCA type				
Intravenous	1		1	
Epidural	1.032 (0.818–1.303)	0.789	1.092 (0.859–1.388)	0.471
Age	1.009 (1.002–1.015)	0.007	1.012 (1.005–1.020)	0.001
Sex				
Male	1		1	
Female	0.961 (0.815–1.133)	0.637	1.014 (0.834–1.232)	0.893
Height	0.997 (0.987–1.007)	0.545	0.981 (0.954–1.010)	0.202
Weight	1.002 (0.994–1.009)	0.671	1.005 (0.996–1.014)	0.288
Comorbidities				
HTN	1.116 (0.935–1.333)	0.225	0.974 (0.800–1.185)	0.790
DM	1.114 (0.898–1.383)	0.325	0.975 (0.772–1.231)	0.829
Cancer stage				
I	1		1	
II	1.364 (0.965–1.927)	0.078	1.330 (0.936–1.890)	0.112
III	1.867 (1.378–2.528)	< 0.0001	1.733 (1.266–2.372)	< 0.001
Tumor histology				
AWD	1			
AMD	0.876 (0.693–1.106)	0.266		
APD	0.910 (0.724–1.143)	0.417		
Mucinous	1.081 (0.688–1.699)	0.735		
SRC	0.884 (0.690–1.133)	0.330		
Other	0.861 (0.544–1.365)	0.525		
Lymphovascular invasion	0.973 (0.840–1.126)	0.714		
Resection method				
Subtotal	1		1	
Total	1.302 (1.115–1.520)	0.001	1.308 (1.112–1.538)	0.001
Anesthetic				
Desflurane	1.140 (0.919–1.415)	0.233		
Enflurane	0.474 (0.273–0.824)	0.008	0.603 (0.340–1.072)	0.085
Isoflurane	1.050 (0.887–1.243)	0.569		
Sevoflurane	0.980 (0.841–1.142)	0.794		
N2O	0.747 (0.590–0.945)	0.015	0.823 (0.643–1.052)	0.120
Propofol 1%	1.115 (0.934–1.331)	0.228		
Thiopental sodium	0.894 (0.746–1.072)	0.226		
Other perioperative drugs				
Aspirin	1.031 (0.257–4.129)	0.966		
NSAIDs	0.905 (0.783–1.045)	0.174		
Ephedrine	1.106 (0.946–1.292)	0.208		
Phenylephrine	1.442 (1.045–1.989)	0.026	1.255 (0.893–1.763)	0.192
Norepinephrine	2.953 (1.219–7.153)	0.016	1.866 (0.685–5.082)	0.222
Esmolol	1.380 (0.990–1.924)	0.058		
Labetalol	1.222 (0.673–2.221)	0.501		
Nicardipine	1.570 (0.940–2.624)	0.085	1.454 (0.859–2.460)	0.163
Intraoperative fluid				
Colloid	1.076 (0.892–1.297)	0.444		
Packed RBC	1.097 (0.735–1.637)	0.650		

CI = Confidence Interval PCA = patient controlled analgesia, HTN = hypertension, DM = diabetes mellitus, RBC = red blood cells.

**Table 3:** Univariate and multiple regression analysis of variables associated with mortality after open gastrectomy for gastric cancer

	Univariate Analysis		Multiple Analysis	
	Hazard Ratio (95% CI)	*P*-value	Hazard Ratio (95% CI)	*P*-value
PCA type				
Intravenous	1		1	
Epidural	0.818 (0.655–1.021)	0.075	0.695 (0.429–1.125)	0.138
Age	1.007 (1.001–1.013)	0.030	1.015 (1.003–1.027)	0.016
Sex				
Male	1		1	
Female	1.110 (0.945–1.304)	0.205	0.905 (0.590–1.389)	0.649
Height	0.990 (0.981–0.999)	0.028	0.989 (0.964–1.014)	0.385
Weight	0.994 (0.986–1.001)	0.086	0.997 (0.982–1.012)	0.689
Comorbidities				
HTN	1.029 (0.878–1.207)	0.720	0.753 (0.555–1.021)	0.068
DM	1.130 (0.933–1.369)	0.210	1.148 (0.801–1.645)	0.452
Cancer stage				
I	1		1	
II	1.338 (1.016–1.762)	0.038	1.745 (1.146–2.657)	0.009
III	1.717 (1.367–2.156)	< 0.0001	2.193 (1.476–3.257)	< 0.001
Tumor histology				
AWD	1			
AMD	0.949 (0.663–1.358)	0.774		
APD	1.220 (0.860–1.731)	0.265		
Mucinous	0.963 (0.591–1.568)	0.879		
SRC	1.157 (0.795–1.684)	0.447		
Other	1.103 (0.651–1.867)	0.715		
Lymphovascular invasion	1.198 (1.024–1.401)	0.025	0.865 (0.657–1.139)	0.302
Resection method				
Subtotal	1		1	
Total	1.350 (1.165–1.565)	< 0.0001	1.473 (1.155–1.877)	0.002
Anesthetic				
Desflurane	1.149 (0.928–1.425)	0.203		
Enflurane	0.418 (0.235–0.745)	0.003	0.549 (0.297–1.012)	0.055
Isoflurane	0.921 (0.782–1.083)	0.318		
Sevoflurane	1.118 (0.964–1.296)	0.140		
N2O	0.761 (0.610–0.950)	0.016	1.087 (0.824–1.433)	0.556
Propofol 1%	1.017 (0.864–1.198)	0.837		
Thiopental sodium	1.059 (0.895–1.252)	0.505		
Other perioperative drugs				
Aspirin	1.535 (0.493–4.775)	0.459		
NSAIDs	0.846 (0.729–0.981)	0.027	1.074 (0.838–1.376)	0.573
Ephedrine	1.151 (0.993–1.334)	0.063	1.076 (0.836–1.385)	0.569
Phenylephrine	1.455 (1.122–1.887)	0.005	2.070 (1.169–3.664)	0.013
Norepinephrine	1.740 (0.982–3.081)	0.058	2.638 (1.040–6.690)	0.041
Esmolol	1.252 (0.922–1.701)	0.150		
Labetalol	1.904 (1.099–3.299)	0.022	10.623 (1.31–86.135)	0.027
Nicardipine	1.366 (0.865–2.156)	0.181		
Intraoperative fluid				
Colloid	0.859 (0.724–1.020)	0.083	0.758 (0.575–0.999)	0.050
Packed RBC	1.300 (0.929–1.818)	0.126		

CI = Confidence Interval PCA = patient controlled analgesia, HTN = hypertension, DM = diabetes mellitus, RBC = red blood cells.

In our PSM analysis, there was no difference in recurrence and mortality between the two groups (*P* = 0.623 and *P* = 0.695, respectively) (Table 4).

**Table 4:** Association between PCA type and post-surgery prognosis in 1-to-1 propensity score-matched gastric cancer patients that underwent open gastrectomy

Outcome	IV PCA (*n =* 373)	Epidural PCA (*n =* 373)	Hazard Ratio (95% CI)	*P*-value
Recurrence			1.098 (0.756–1.596)	0.623
No	292 (78.3)	304 (81.5)		
Yes	81 (21.7)	69 (18.5)		
Mortality			0.855 (0.391–1.869)	0.695
No	283 (75.9)	291 (78.0)		
Yes	90 (24.1)	82 (22.0)		

Values are *n* (%) of patients CI = Confidence Interval, PCA = patient controlled analgesia.

## DISCUSSION

The perioperative period during which the patient is in the hands of the anesthesiologist is considered as a narrow window of opportunity where outcome after surgery may be affected by the choice of drugs and method of anesthesia and/or analgesia. [26] While surgical resection of the primary tumor can be ultimately curative, it also exposes the patient to an immunologically vulnerable period during which the patient is relatively more prone to undetectable residual disease or micrometastasis. [27] Although it is still largely under debate, neuraxial techniques have been suggested to be able to improve prognosis after cancer surgery. However, the findings of the present retrospective analysis show otherwise, where we failed to find any difference in recurrence or mortality after curative surgery for gastric cancer between patients that received either IV or epidural PCA for postoperative analgesia.

Proposed mechanisms through which regional anesthesia and analgesia may influence cancer outcome can be summarized into “immunomodulation” and “anti-inflammation”. The immunomodulatory and anti-inflammatory effects of regional anesthesia and analgesia can be thought of as the combined output of several different mechanisms which include the attenuation of sympathetic nervous system stimulation in response to surgical stress and postoperative pain, the sparing of the need of opioids and therefore the immunosuppressant effects of such drugs, and the direct cytotoxic and anti-inflammatory effects of amide local anesthetics such as ropivacaine which was used in all of the patients included in our analysis. [27, 28].

However, whether the aforementioned immunomodulatory and anti-inflammatory effects of neuraxial techniques actually translate into a positive effect on cancer outcome is not clear, and the existing evidence present conflicting results in various types of cancer. [27] This is also true for gastric cancer, where epidural anesthesia and/or analgesia are commonly used. A rare randomized trial that studied the effect of epidural analgesia on cancer prognosis after major abdominal surgery concluded that cancer recurrence and mortality rates were not different between patients that received general anesthesia combined with epidural anesthesia and analgesia, compared to those that received general anesthesia with opioid analgesia. [16] However, the proportion of patients with gastric cancer was lower than 15% overall, and approximately 50% of patients were those with colorectal cancer. It is difficult to generalize its results mainly due to the fact that tumor biology varies significantly between organs. Also, while this study is significant in that it is probably the only randomized trial to date that studied the effect of epidural block with follow-up results up to 5 years, the analysis itself was a retrospective review of follow-up data from a previous RCT.

Although gastric cancer is the fifth most common malignancy and the third leading cause of cancer death worldwide, due to its relative uncommonness in Northern America and Europe, studies conducted in gastric cancer patients are lacking in comparison to studies in colorectal, breast, prostate and ovarian cancer. As of today, the majority of existing retrospective studies report no association between epidural anesthesia and/or analgesia and gastric cancer prognosis. Hiller et al. [4] reported an association between postoperative epidural analgesia and benefit on cancer recurrence and survival following surgery for esophageal, but not gastric cancer in 2014. A similar study by Cummings et al. [25] also published in the same year reported no association between postoperative epidural analgesia and reduced recurrence or improved survival after resection in gastric cancer patients. These results somewhat dampened the earlier enthusiasm towards the possible ability of perioperative epidural techniques to modulate cancer recurrence. A more recent retrospective study that compared combined epidural and general anesthesia with general anesthesia alone, reported no difference in long-term survival in gastric cancer patients [14]. Interestingly, in contrast to the aforementioned negative reports, a recent retrospective study of 4,218 patients by Wang et al. [15] concluded that epidural anesthesia combined with general anesthesia and postoperative epidural PCA may be associated with improved survival after resection for gastric cancer. However, despite having the merit of a largest sample size to date, this study only looked at overall survival, but not cancer recurrence after surgery. Also, the majority of patients included in this study were of advanced stage (Stage I = 28.9%, Stage II = 4.3%, Stage III = 66.9%), which may have affected their results. The patients of the present study showed a relatively more even distribution among cancer stages (Stage I = 53.8%, Stage II = 18.1%, Stage III = 28.1%) compared to the previous retrospective analysis.

Our study is not the first retrospective analysis to report negative association between epidural analgesia and gastric cancer prognosis. However, the present study seems to have great merit in that it is the first to include a very homogeneous group of patients from a reliable database of a high-volume center. Overlooking the importance of the nature of the surgical procedure as well as surgical technique can easily lead to incorrect interpretations in cancer patients. By including a homogeneous group of patients that underwent curative surgery, the present study was able to analyze the effect of epidural analgesia on recurrence after complete resection. Moreover, standardized extended (D2) lymphadenectomy was employed as a uniform surgical technique in all patients that were included in our analysis. Compared to limited (D1) lymphadenectomy, the D2 procedure has been proven to be associated with lower recurrence and gastric-cancer-related mortality, and is currently recommended as the surgical approach of choice for patients with curable gastric cancer. [29] The good quality database and further PSM analysis that was used in the present study has been able to increase the reliability of our results. Whether epidural analgesia is able to affect length of survival or mortality in patients undergoing palliative or non-curative surgery requires future studies.

Despite the negative results of our study, it is difficult to conclude that regional techniques are not related to outcome after gastrectomy. One may suspect that isolated use of postoperative epidural analgesia without combining intraoperative epidural anesthesia is insufficient to induce effective immunomodulatory or anti-inflammatory effects. It seems most important to acknowledge that epidural analgesia is among the many different factors associated with perioperative anesthesia care that have been suggested to have effect on tumor progression and cancer prognosis. [27] It would be a futile attempt to improve cancer prognosis with a single drug or procedure, and therefore clinicians should be able to achieve good analgesia, and ameliorate perioperative stress and inflammation overall. [30]

The association between the use of norepinephrine and phenylephrine and greater hazard for mortality is most likely due to greater hemodynamic instability and hence the need for more vasopressors in patients with poorer general condition. Due to the retrospective design of the present analysis, we cannot conclude whether such drugs are definite causes of mortality or whether they are simply markers of worse clinical outcome. Among other perioperative drugs of interest, both aspirin [31, 32] and NSAIDs [33] showed no association with reduced recurrence or mortality. Interestingly, the use of labetalol, a non-selective β adrenergic antagonist, was found to be associated with greater mortality after gastrectomy which is in contrary to previous reports. [34–36] However, the confidence interval was very wide, which indicates that our sample size was too small to draw conclusions in this aspect.

The major limitation of the present study is its retrospective nature, and thus its susceptibility to chance, bias and other confounding factors. However, we were able to include a relatively large number of patients from a single center, high-quality database in our analysis, and also performed propensity score matching with the available covariates to overcome potential selection bias somewhat. Although definitive data can only be provided by future RCTs, the results after propensity score matching in the present study should be able to add valuable insight to this debatable issue. Another limitation is that it is unclear how long the epidural catheters and PCA devices were maintained, and whether postoperative epidural analgesia was prematurely discontinued in patients included in the present analysis. Of note, it has been routine practice to start epidural PCA machines at the end of surgery and maintain infusion for 3 days in patients undergoing gastrectomies at our hospital. Also, intraoperative epidural infusions are rarely used. Therefore the results of the present study should be interpreted as a comparison between postoperative analgesic methods, and not as anesthesia. However, intermittent or complete discontinuation of epidural infusions due to hypotension or side effects of opioids are not uncommon in postoperative patients, and the absence of this data may have affected the results of our analysis.

In conclusion, postoperative epidural PCA does not seem to be associated with reduced recurrence or lower mortality in patients undergoing open curative gastrectomy for gastric cancer. Epidural analgesia should be employed as part of a comprehensive perioperative management plan, but should not be relied on as a beneficial factor for the prognosis after curative surgery in gastric cancer patients.

## MATERIALS AND METHODS

### Study population and design

This study is a retrospective analysis of patients that underwent open gastrectomy for gastric cancer with either IV or epidural PCA for postoperative pain control. The study protocol was approved by the Institutional Review Board and Hospital Research Ethics Committee of Severance Hospital, Yonsei University Health System (IRB #4-2017–0392). The data collected for this study was from the electrical medical records of patients that underwent surgery between November 2005 and December 2010 at a single institution. Patients that received pre-operative chemotherapy or those that underwent non-curative surgery, cases that started as laparoscopic procedures but converted to laparotomy, electronic charts with unclear method of postoperative analgesia, and mortality cases that occurred within 30 days after surgery were excluded from analysis.

Retrieved demographic data of the patients included age, sex, height, weight, and comorbidities such as hypertension (HTN), diabetes mellitus (DM), pulmonary diseases, etc. In addition to type of PCA, cancer stage, extent of gastrectomy, type of anesthetic, common intraoperative drugs, and intraoperative colloid administration and red blood cell (RBC) transfusion were also analyzed as variables possibly associated with the prognosis of gastric cancer after open gastrectomy.

### Statistical analysis

The primary outcome of this study was to compare the recurrence rate of gastric cancer between the two postoperative analgesia methods. Demographic and perioperative characteristics were analyzed using the Student’s *t-*test for continuous variables and the chi-square test for categorical variables. Potential factors affecting cancer recurrence and mortality after gastrectomy were analyzed by the Cox proportional hazards model, and the risk of each variable was calculated as hazard ratio (HR) and 95% confidence intervals (CIs). After screening for potentially significant variables with univariate analysis, multiple Cox regression analysis was done by including factors with *P* values < 0.2. Overall survival rates during the study period, and difference between survival curves according to PCA route were examined with the Kaplan–Meier log-rank survival analysis method. Propensity score matching (PSM) was done to reduce selection bias, and confounders used for PSM included age, sex, body mass index, presence of DM and HTN. After 1:1 matching with propensity scores calculated with logistic regression analysis with aforementioned confounding factors, the risk of recurrence and mortality with each type of PCA was analyzed with conditional Cox proportional hazard regression. *P* values less than 0.05 were considered as statistically significant. All analyses were done with SAS version 9.4.

## Abbreviations

CI: confidence interval
DM: diabetes mellitus
HR: hazard ratio
HTN: hypertension
IRB: institutional review board
IV: intravenous
PCA: patient controlled analgesia
PSM: propensity score matching
RBC: red blood cell
RCT: randomized controlled trial
